# Plane inclinations: A critique of hypothesis and model choice in Barbi et al

**DOI:** 10.1371/journal.pbio.3000048

**Published:** 2018-12-20

**Authors:** Saul Justin Newman

**Affiliations:** Research School of Biology, The Australian National University, Acton, ACT, Australia

## Abstract

This study highlights how the mortality plateau in Barbi and colleagues can be generated by low-frequency, randomly distributed age-misreporting errors. Furthermore, sensitivity of the late-life mortality plateau in Barbi and colleagues to the particular age range selected for regression is illustrated. Collectively, the simulation of age-misreporting errors in late-life human mortality data and a less-specific model choice than that of Barbi and colleagues highlight a clear alternative hypothesis to explanations based on evolution, the cessation of ageing, and population heterogeneity.

## Abstract 2

This Formal Comment raises fundamental issues regarding the apparent late-life mortality plateau proposed in Science by Barbi et al. 2018, and on the nature of documentary birth and death certificate evidence in general.

## Text

The authors of Barbi and colleagues [1] have proposed that late-life plateaus require structural and evolutionary explanations, including the potential contribution of population heterogeneity, to explain a ‘real’ mortality plateau [1]. However, Barbi and colleagues correctly highlight an alternative hypothesis: the potential for age misreporting and cohort blending errors to generate artefactual late-life plateaus. Despite raising this hypothesis, the effect of low-frequency age-misreporting errors was not actively addressed.

**Fig 1:**
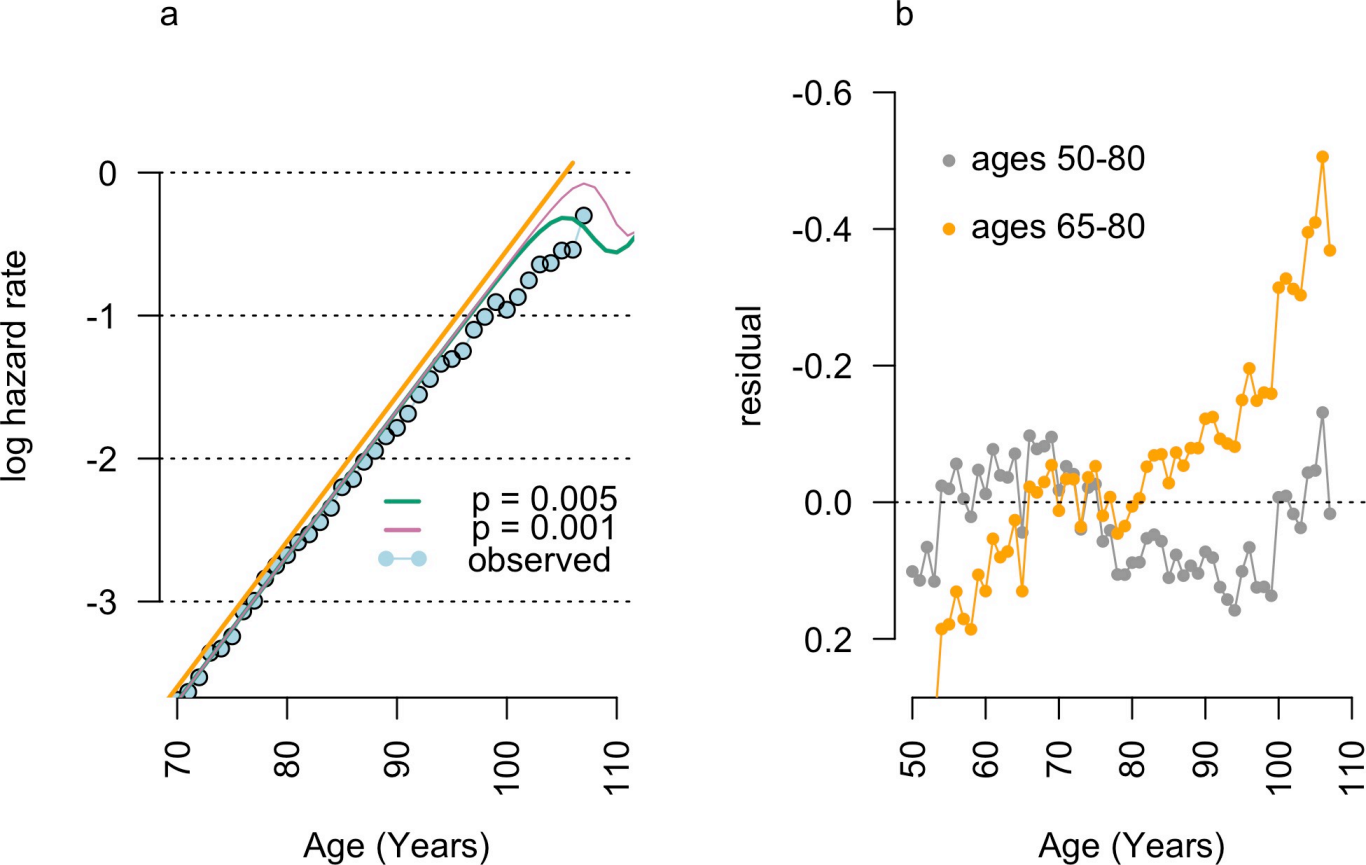
Generation of late-life mortality plateau by random errors. The introduction of symmetrically distributed age-coding errors (a) into the log-linear model (orange line) fit as a 'best estimate’ to the 1904 cohort data in Barbi and colleagues [1] generates late-life mortality patterns (green and pink lines) similar to observed data (blue points) [4]. Residuals from observed data (b) illustrate the skewed residuals of this model compared with more representative regressions of hazard rates, such as the ages 50–80 model shown (grey).

Therefore, late-life plateaus observed in the 1904 cohort were compared to the effect of simulated, low-frequency age-reporting errors introduced into log-linear models of mortality fitted to the age range of 65–80 used in Barbi and colleagues [1]. Following Newman (this issue) [2], random symmetrically distributed five- and 10-year age-coding errors were seeded into these synthetic cohorts at age 50 with a probability ranging from *p* = 10^−3^ to *p* = 10^−6^ (Fig 1A; mean = 0; SD = 7.9; S1 Code). These error rates were selected as hypothetical best-case scenarios, exceeding the accuracy of clinical trial data entry [3].

Age-misreporting errors generated late-life plateaus at frequencies below 1 in 500 when introduced into the ‘best-estimate’ 65- to 80-years-old model presented by Barbi and colleagues (Fig 1A and 1B). Furthermore, hazard rates resulting from these random errors closely resembled the late-life plateau of Barbi and colleagues (Fig 1A) and required no biological or evolutionary explanation.

**Fig 2:**
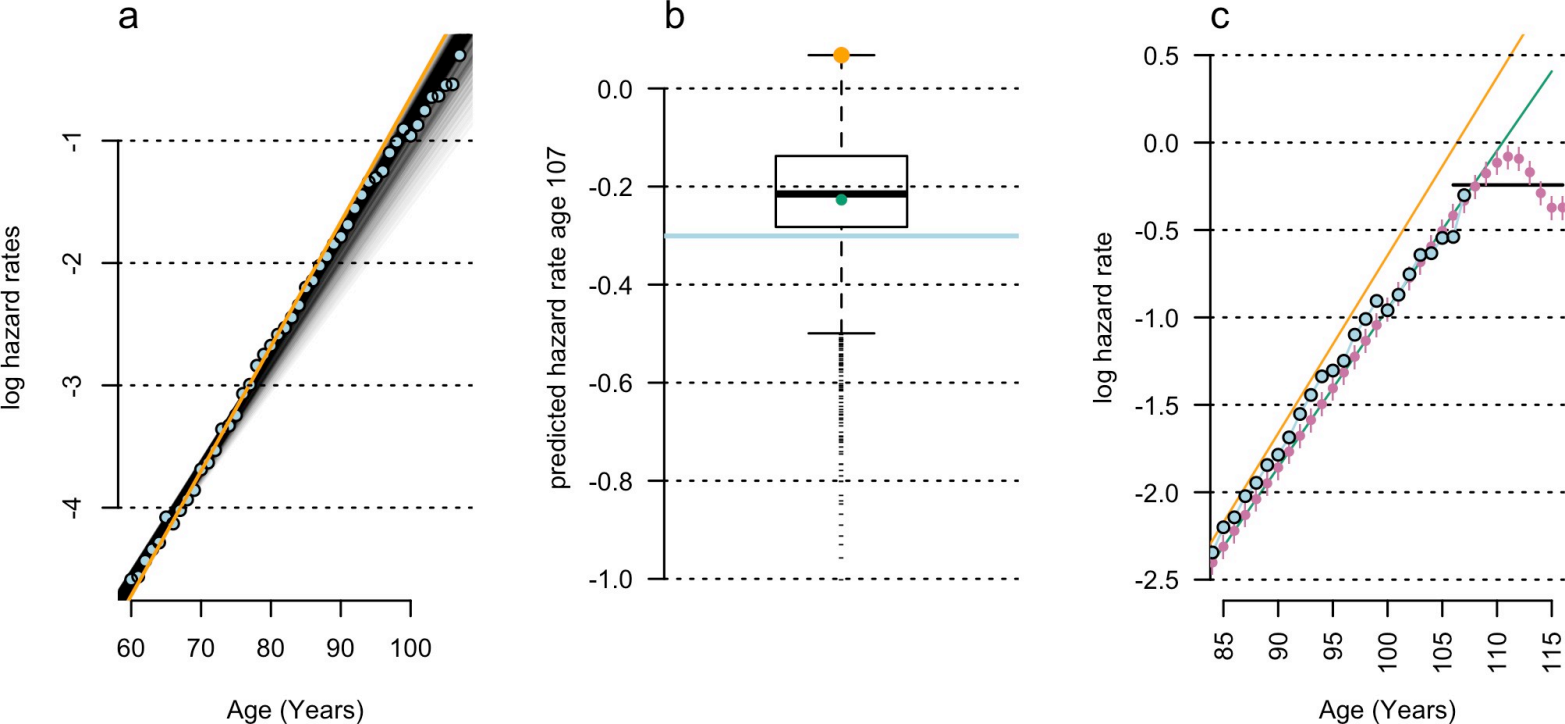
Effect of model selection on the size of apparent mortality plateaus. (a) Observed hazard rate data (blue) from Barbi and colleagues [1], fitted by log-linear hazard rate regressions for 861 diverse age ranges (grey lines). The Barbi and colleagues [1] age range (orange) produces the largest late-life mortality plateau and (b) produces the greatest overestimate of observed data (orange cross) at advanced ages (blue line, age 105 shown). Seeding random errors into other representative models—e.g., the 50- to 80-years-old regression (green point in (b), green line in (c))—produces (c) late-life mortality deceleration (pink points; *p* = 2 × 10^−4^) and constant hazard-rate regressions (black line) past age 105.

The apparent size of the late-life plateau in Barbi and colleagues [1] is characterized in comparison to a ‘best-estimate trajectory’ model, fitted to data from a specific age range of 65–80 years. The poor fit of this mid-life model to late-life data is used as justification for fitting separate late-life models. However, the choice of age range used to fit log-linear models has a large effect on the size and existence of late-life mortality plateaus (Fig 2).

Barbi and colleagues selected an age range of 65–80 years [1]. This modelling choice was compared to log-linear models fitted to mortality data using the 1904 Italian cohort data [4] used by Barbi and colleagues [1], fitted to all possible age range combinations starting between ages 45–65 inclusive and ending between ages 70–107 inclusive. Of the 861 age-range combinations tested, the model selected by Barbi and colleagues generated the single largest late-life mortality plateau (Fig 2B and 2C). This model choice also provided the worst fit of mid-life (age 50) and late-life mortality patterns (Fig 2B).

Re-fitting these models to other age ranges reduced the apparent deviation of late-life data from mid-life patterns of mortality (Fig 1) and reduced or eliminated the late-life plateau in these data. Furthermore, models fitted to alternative age ranges have a greatly reduced threshold for age-misreporting errors to cause late-life mortality deceleration and plateaus. For example, artificial mortality plateaus are produced by age-misreporting rates below 1 in 10,000 if a log-linear model fitted to ages 50–80 is used (Fig 2C). If these error patterns are fitted by log-linear models using the same parameters as Barbi and colleagues [1], constant-hazard–mortality ‘plateaus’ are produced (Fig 2C).

Simulated error rates were low compared to observed rates of manual entry errors. For example, double-entry of clinical trial data has error rates of *p* = 10^−3^ [3]. The pattern of late-life mortality deceleration shown in Fig 1A (pink line) results from random errors generated at half this rate. However, birth certificate data in the Barbi and colleagues data constitute hand-written records generated by a cohort with 32% literacy rates [5] and 9.6 months of education on average [6]. For error-generated late-life plateaus to be excluded from the data in Barbi and colleagues, this cohort would have to achieve error rates 2- to 1,000-fold lower than that observed in clinical trials. This seems unlikely.

Finally, claims by Barbi and colleagues that age data are validated by documents, and therefore ‘real’, should be viewed in the context of previously validated longevity claims. For example, Carrie White successfully claimed to be the world’s oldest and then second-oldest person. Documents certifying her age endured global scrutiny for 24 years. Her record was verified and accepted by the Gerontology Research Group and the Guinness World Records Book.

The Carrie White record was retracted in 2012 after it was shown to be the result of a clerical error that inflated her apparent age from 102 to 116. A single age-coding error by a mental institution worker was copied to all later documents and validated. Absent the survival of this otherwise obscure document, the Carrie White record would stand as a document-validated supercentenarian.

All supercentenarian data in Barbi and colleagues are susceptible to similar, but undetected, errors. In similar Gerontology Research Group data, 8% of all supercentenarian cases were found to be errors [7], and the potential for many other undocumented cases remains. As such, asserting that supercentenarian data are ‘clean data’ because they have passed document-based validation is unfounded.

The capacity for data entry and age inflation errors provides a sufficient model to explain late-life mortality patterns observed by Barbi and colleagues without requiring a cessation of ageing, population heterogeneity, or evolutionary theories. This suggests the late-life mortality plateau observed was a result of errors, not biology.

## Supporting information

S1 Code Code underlying the findings. (TXT)

## References

[1] Barbi E, Lagona F, Marsili M, Vaupel JW, Wachter KW. The plateau of human mortality: Demography of longevity pioneers. Science. 2018;360: 1459–1461. 10.1126/science.aat3119 29954979 PMC6457902

[2] Newman SJ. Errors as a primary cause of late-life mortality deceleration and plateaus. PLoS Biol. 2018;16(12):e2006776 10.1371/journal.pbio.2006776 PMC6301557 30571676

[3] Reynolds-Haertle RA, McBride R. Single vs. Double data entry in CAST. Control Clin Trials. 1992;13: 487–494. 10.1016/0197-2456(92)90205-E 1334820

[4] Human Mortality Database [Internet]. University of California, Berkeley (USA), and Max Planck Institute for Demographic Research (Germany).; 2013 [cited 2015 Dec 7]. Available: www.mortality.org

[5] Broadberry SN, O’Rourke KH. The Cambridge Economic History of Modern Europe: 1700–1870 The Cambridge Economic History of Modern Europe. Cambridge: Cambridge University Press; 2010 10.1017/CBO9780511794834

[6] Timmer M, Baten J, Rijpma A, Smith C, van Zanden JL, Mira d’Ercole M. How Was Life?: Global Well-being since 1820. Paris: OECD publishing; 2014.

[7] Gerontology Research Group [Internet]. [cited 21 Oct 2016]. Available: http://www.grg.org/SC/SCindex.html

